# Bulky DNA adducts in human sperm associated with semen parameters and sperm DNA fragmentation in infertile men: a cross-sectional study

**DOI:** 10.1186/1476-069X-12-82

**Published:** 2013-09-30

**Authors:** Guixiang Ji, Lifeng Yan, Shengmin Wu, Jining Liu, Lei Wang, Shenghu Zhang, Lili Shi, Aihua Gu

**Affiliations:** 1 Nanjing Institute of Environmental Sciences/Key Laboratory of Pesticide Environmental Assessment and Pollution Control, Ministry of Environmental Protection, Nanjing 210042, China; 2 State Key Laboratory of Reproductive Medicine, Institute of Toxicology/Key Laboratory of Modern Toxicology of Ministry of Education, School of Public Health, Nanjing Medical University, Nanjing 210029, China

**Keywords:** Polycyclic aromatic hydrocarbons (PAHs), Bulky DNA adducts, Semen quality, Sperm integrity, Epidemiology

## Abstract

**Background:**

DNA adducts are widely used marker of DNA damage induced by environmental pollutants. The present study was designed to explore whether sperm polycyclic aromatic hydrocarbon-DNA adducts were associated with sperm DNA integrity and semen quality.

**Methods:**

A total of 433 Han Chinese men were recruited from an infertility clinic. Immunofluorescence was applied to analyze sperm PAH-DNA adducts. Sperm DNA fragmentation was detected by terminal deoxynucleotidyl transferase (Tdt)-mediated dUTP nick end labelling (TUNEL) assay.

**Results:**

After adjustment for potential confounders using linear regression, sperm PAH-DNA adducts were negatively associated with sperm concentration, total sperm count, sperm motility, and curvilinear velocity (VCL). In addition, a positive relationship between sperm PAH-DNA adducts and sperm DNA fragmentation was found.

**Conclusions:**

Our findings suggested an inverse association between sperm PAH-DNA adducts and semen quality, and provided the first epidemiologic evidence of an adverse effect of PAH-DNA adducts on sperm DNA integrity.

## Background

Polycyclic aromatic hydrocarbons (PAHs) are widespread pollutants in the environment that are generated by the incomplete burning of organic substances. Some reports showed high detection rates of PAHs metabolites among different races and genders, reflecting ubiquitous exposure to the parent compounds in the general population [1-3]. In China, due to the conventional eating habits that involve heavily fried, roasted, or grilled foods and the rapid increase of automobile and industrial production, the general population has more opportunities to be exposed to PAHs compared to other nations. It has been reported that the creatinine-adjusted 1-OHP metabolites levels in Nanjing region is 16-fold higher than in U.S. populations [4]. This result suggests Chinese adult males are highly exposed to PAHs in the environment, so the potential health hazard of PAHs deserve more attention in China.

PAHs bind covalently to DNA to form DNA adducts which are the indicator of DNA damage and are considered to be human mutagens and carcinogens [5]. In the reproductive system, several animal studies have suggested possible associations between PAHs exposure and reproductive function [6-8]. However, very limited epidemiologic data exist on the potential effects of PAHs exposure on human reproductive functions. In humans, several studies suggested the relationship between air pollution exposure to PAHs and increased sperm DNA damage [9,10].

Some recent studies have assessed the non-occupational exposure to PAHs by measuring the metabolite 1-hydroxypyrene (1-OHP) in urine, and found suggestive associations with altered semen quality and sperm DNA damage [4,11,12]. However, urinary 1-OHP represents the exposure within the last 24 hours, whereas DNA adducts indicate the accumulated exposure over months [13]. So 1-OHP has frequently been used to assess the occupational exposure, and PAH-DNA adducts are considered as the valid marker for evaluating low level environmental exposures. In addition, it has been implicated that measuring PAH-DNA adducts in human tissues can provide integrated information about genotoxic effects of PAHs mixtures, since it takes into account individual differences in exposure, absorption, distribution, metabolic activation and detoxification of PAHs in the body as well as cell turnover and repair of DNA damage [14-16].

In the present study, we measured PAH-DNA adducts in human sperm by immunofluorescence assay and detected their potential relations with semen quality. In addition, we extended previous human studies on sperm DNA damage measured by TUNEL assay by including a larger sample of men. Investigation of environmental impacts on sperm DNA damage is important since growing and consistent evidence shows that DNA damage in human sperm appears as a risk factor for adverse clinical outcomes including poor semen quality, low fertilization rates, impaired pre-implantation development, and an increased risk of morbidity in the offspring [17-19].

## Methods

### Subjects and sample collection

Study subjects were candidates seeking treatment in the Center of Clinical Reproductive Medicine (affiliated hospitals of Nanjing Medical University) between April 2005 and March 2007 (NJMU Infertile Study). The protocol and consent form were approved by the Institutional Review Board of Nanjing Medical University prior to the study. All activities involving human subjects were done under full compliance with government policies and the Helsinki Declaration. A complete physical examination, including height and weight, was performed, and a questionnaire was used to collect information, including personal background, lifestyle factors, occupational and environmental exposures, genetic risk factors, sexual and reproduction status, medical history and physical activity. Men with abnormal sexual and ejaculatory functions, immune infertility, semen non-liquefaction, medical history of risk factors for infertility (e.g. varicocele, postvasectomy or orchidopexy) and receiving treatment for infertility (e.g. hormonal treatments) were excluded from the study. Men with other known factors related to male infertility, such as genetic disease, infection, occupational exposure to PAHs or other agents suspected to be associated with male reproduction were also excluded. Furthermore, to avoid spermatogenesis impairment caused by Y chromosome microdeletions, we excluded subjects with Y chromosome microdeletions of azoospermia factor region [20]. A single spot semen samples were collected by masturbation after at least 2 days of sexual abstinence. Totally, 433 infertility males were included in the present study.

### Semen quality analysis

Semen samples were obtained in a private room by masturbation into a sterile wide-mouth and metal-free glass container after a recommended 2-day sexual abstinence. After liquefaction at 37°C for 30 min, conventional semen analysis including semen volume, sperm concentration, sperm number per ejaculum, and sperm motility was performed by using the computer assisted semen analysis (CASA, WLJY 9000, Weili New Century Science and Tech Dev.). Setting parameters and the definition of measured sperm motion parameters for CASA were established by the manufacturer. Percent motile sperm was defined as WHO grade “A” sperm (rapidly progressive with a velocity ≥25 μm/sec) plus grade “B” sperm (slow/sluggish progressive with a velocity ≥5 μm/sec but <25 μm/sec) [21]. Of nine CASA variables that were measured, only three were chosen [straight-line velocity (VSL, μm/s), curvilinear velocity (VCL, μm/s) and linearity (LIN = VSL/VCL × 100, %)] for inclusion in the present analysis due to a high degree of dependence between several of the measures. Observation and counting in the semen analysis were automatic, and the fertility status and exposure levels of the men whose samples were being assessed were blinded to avoid bias.

### DNA fragmentation analysis

The Tdt-mediated dUTP nick-end labeling (TUNEL) assay has been shown to be a feasible and sensitive way to detect DNA fragmentation [22]. We used the APO-DIRECT kit (BD Biosciences PharMingen, San Diego, CA, USA) according to the manufacturer’s protocol. Briefly, sperm were washed and resuspended in 2% paraformaldehyde for 30 min at room temperature. After being rinsed with PBS, samples were resuspended in permeabilisation solution (0.2% Triton X-100, 0.1% sodium citrate) for 10 min on ice. Fifty millilitres of TUNEL reagent was then added to the sample. For each batch, samples that were not treated with the Tdt enzyme were used as negative controls, and samples treated with DNase I were included as a positive control. After incubation for 1 h at 37°C, samples were analysed immediately by flow cytometry (FACSCalibur; BD Biosciences Pharmingen, San Diego, CA, USA).

### PAH-DNA adducts determination: Immunofluorescence staining

PAH-DNA adducts were determined by an indirect immunofluorescence as previously described [23] using BPDE-DNA (5D11) monoclonal antibody (sc-52625; Santa Cruz). The level of adducts was estimated by the intensity of FITC fluorescence, which detected by the FACSCalibur flow cytometer (BD Biosciences Pharmingen). Mean fluorescence intensity (MFI) was calculated on a logarithmic scale. For each batch, a negative control with normal mouse IgG instead of the 5D11 was included.

### Statistical analysis

The statistical analyses were performed with Statistical Analysis System software (version 9.1.3, SAS Institute, Cary, NC). All tests were two-sided and the significance level was set at *P* < 0.05. Linear regression model was conducted to explore the associations between semen parameters, sperm DNA integrity and sperm PAH-DNA adducts. In order to identify the exposure-response relations and the possible thresholds of exposure below or above which effects were diminished, participants were divided into four arbitrary groups by sperm PAH-DNA adducts levels that represented a reasonable trade-off between exposure contrast and group size (30.2-42.5, 42.8-52.9, 53.1-77.2 and 78.2-117.1). *P* values for linear trend were calculated to assess whether there were linear trends in the mean variable values across ranked categories of sperm PAH-DNA adducts levels. Some of the outcome variables, e.g. sperm DNA fragmentation and sperm concentration, total sperm count had somewhat skewed distributions (checked by skewness-kurtosis tests). These variables were transformed using the natural logarithm, whereas all other semen-quality measures, semen volume, sperm motility, VSL, VCL and LIN were modeled untransformed. Age (as a continuous variable), body mass index (as a continuous variable), abstinence time (2–3, 4–5, 6–7 and ≥8 days), smoking and alcohol drinking status (current and former vs. never) were considered as potential confounders for the semen characteristics, and we included them in multivariate models.

## Results

**Figure 1:**
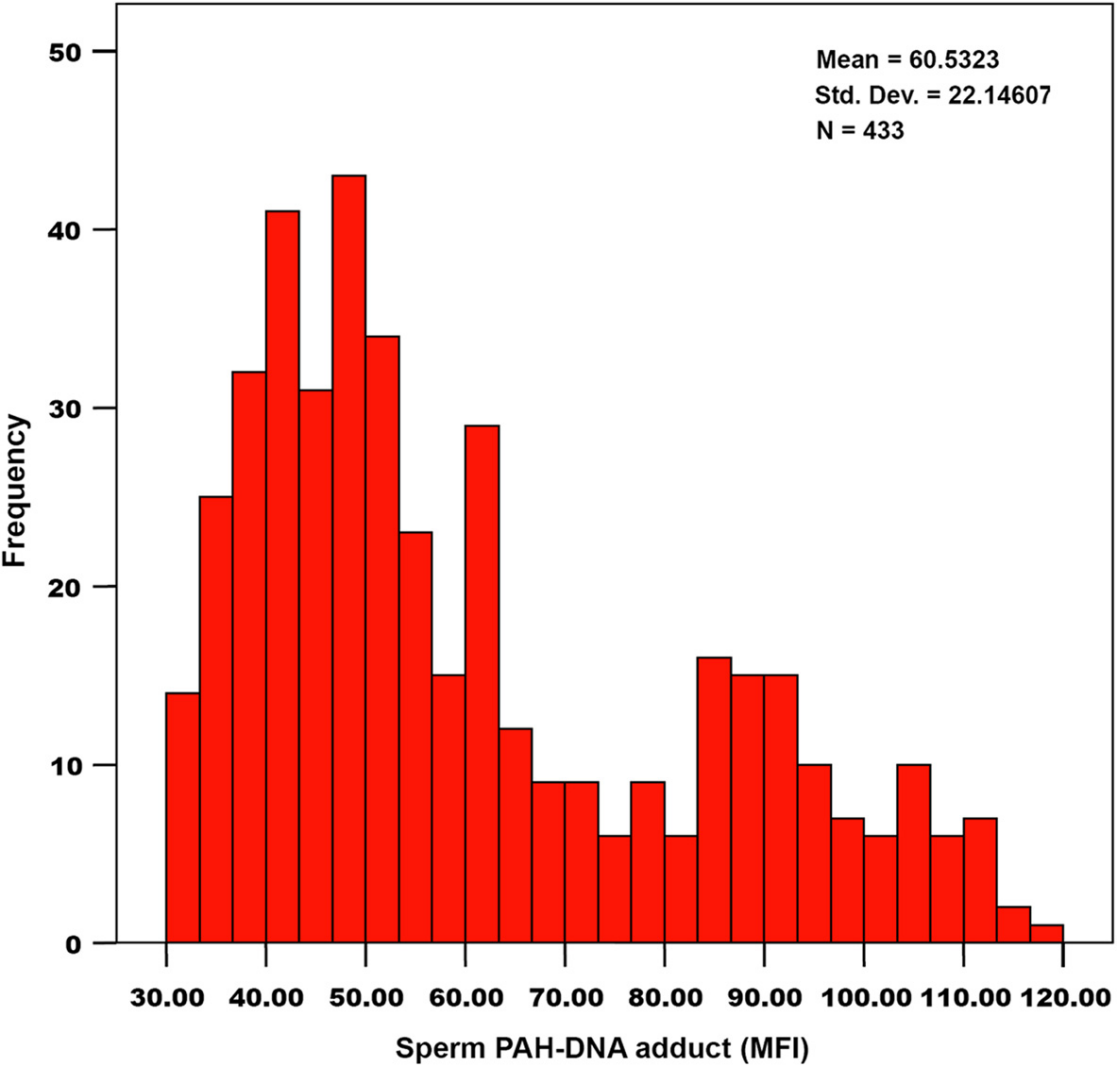
Distribution of sperm PAH-DNA adducts levels in 433 patients.

A total of 433 eligible men (92% participation) provided their semen samples. The levels of sperm PAH-DNA adducts were widely and non-normally distributed (Figure 1). Demographic information and semen parameters were given in Table 1. The mean (± SD) of age, BMI and abstinence time of the 433 subjects were 28.4 ± 3.3 years, 23.3 ± 2.8 kg/m^2^ and 6.0 ± 5.0%, respectively. Forty-two percent of subjects were never smokers, and 49.4% were current smokers (smoked within the past month). The mean of sperm concentration, sperm motility and total sperm count were 72.8 million/ml, 55.3% and 251.7 million. Although the mean values were all larger than the reference values for each semen parameter (WHO, 1999), 63.3% of subjects had values in one or more semen parameters below the WHO reference. The mean of sperm DNA fragmentation detected by TUNEL assay was 18.7%. According to the 30% threshold for TUNEL assay to distinguish between fertile controls and infertile men [24], 21.9% of the patients reached the critical threshold.

**Table 1:** Subjects demographic information and semen parameters and sperm fragmentation (n = 433)

**Characteristic**	**Mean ± SD**	**No (%)**
Age (years)	28.4 ± 3.3	
Smoking status		
Never smoker		184 (42.5)
Current smoker		214 (49.4)
Former smoker		35 (8.1)
Body mass index (kg/m^2^)	23.3 ± 2.8	
Abstinence time (days)	6.0 ± 5.0	
≤ 3		65 (15.0)
4-5		234 (54.0)
6-7		81 (18.7)
≥8		53 (12.2)
Semen parameters		
Sperm concentration (10^6^/ml)	72.8 ± 54.2	
Subjects < 20 million sperm/ml^*a*^		107 (24.7)
Sperm motility (% motile)	55.3 ± 26.1	
Subjects < 50% motile sperm^*a*^		188 (43.4)
Total sperm count (million)	251.7 ± 225.5	
Subjects < 40 million sperm^*a*^		129 (29.8)
Sperm DNA fragmentation (%)	18.7 ± 11.5	
Subjects > 30% TUNEL		95 (21.9)

^*a*^According to WHO (1999).

In the linear regression models, semen quality parameters, sperm motion parameters and sperm DNA fragmentation measures were regressed on categories of sperm PAH-DNA adducts (30.2-42.5, 42.8-52.9, 53.1-77.2 and 78.2-117.1). Compared with men who had the lowest sperm PAH-DNA adducts category, men with the highest sperm PAH-DNA adducts level had a suggestive decline in sperm concentration, sperm count, sperm motility and VCL (Table 2). Trend *P*-values of sperm concentration, sperm count, sperm motility, and VCL were <0.001, <0.001, 0.004 and <0.001, respectively. Aside from suggestively negative associations with these semen parameters, categories of sperm PAH-DNA adducts were also associated with a suggestive increasing trend in sperm DNA fragmentation (*P* for trend <0.001).

**Table 2:** Quartiles of sperm PAH-DNA adducts in relation to semen parameters and sperm DNA fragmentation (n = 433)

**Variables**	**Sperm PAH-DNA adducts**				***P *** **for trend**^**b**^
	**Tertile 1**	**Tertile 2**	**Tertile 3**	**Tertile 4**	
	**(n = 108)**	**(n = 108)**	**(n = 109)**	**(n = 108)**	
**Semen quality parameters**					
Seminal volume (ml)	3.84 ± 1.67	3.53 ± 1.52	3.73 ± 1.47	3.80 ± 1.63	0.697
Concentration (10^6^/ml)^a^	4.14 ± 0.89	3.78 ± 1.08*	3.70 ± 1.14*	3.37 ± 1.44*	<0.001
Sperm count (10^6^/ml)^a^	5.31 ± 1.04	5.03 ± 1.16	4.90 ± 1.24	4.35 ± 1.57*	<0.001
Motility (% motile)	52.35 ± 25.22	47.22 ± 27.44	47.98 ± 19.03	41.16 ± 22.56*	0.004
**CASA motion parameters**					
VSL (μm/s)	36.63 ± 12.49	35.46 ± 10.30	35.24 ± 7.50	33.88 ± 9.90	0.057
VCL (μm/s)	44.38 ± 18.33	42.95 ± 10.62	42.97 ± 11.71	35.85 ± 7.45*	<0.001
LIN (%)	58.88 ± 10.96	57.01 ± 12.28	59.48 ± 9.84	55.74 ± 14.26	0.234
**DNA Fragmentation (%)**^a^	2.34 ± 0.95	2.37 ± 0.99	2.56 ± 1.04	2.84 ± 1.01*	<0.001

^a^ln-transformed.

^b^Adjusted for age, smoking, alcohol drinking, BMI and abstinence time.

*P <0.05 compared with the lowest sperm PAH-DNA adducts tertile (Tertile 1).

The regression coefficients for increasing quartiles of sperm DNA-PAH adducts are showed in Table 3. After adjustment for possible confounders, the highest sperm PAH-DNA adducts group was associated with a suggestive 10.9% decline in sperm motility (95% CI, -17.74 to −4.00) and 8.48% decline in VCL (95% CI, -11.97 to −5.00). However as for the other semen parameters as well as sperm DNA fragmentation, the statistically significant effect estimates for the highest sperm PAH-DNA adducts group were not quite as large (i.e., they were closer to zero).

**Table 3:** **Adjusted**^**a **^**regression coefficients for change in semen parameters and sperm DNA fragmentation associated with sperm DNA-PAH adducts categories**

**Variables**	**Sperm PAH-DNA adducts**			
	**Tertile 1**	**Tertile 2**	**Tertile 3**	**Tertile 4**
	**(n = 108)**	**(n = 108)**	**(n = 109)**	**(n = 108)**
**Semen quality parameters**				
Seminal volume (ml)	Reference	−0.31 (−0.74, 0.13)	−0.18 (−0.62, 0.25)	−0.11 (−0.55, 0.33)
Concentration (10^6^/ml)^b^	Reference	−0.33 (−0.65, -0.01)*	−0.37 (−0.70, -0.04)*	−0.69 (−1.02, -0.36)*
Sperm count (10^6^/ml)^b^	Reference	−0.24 (−0.60, 0.10)	−0.36 (−0.72, 0.01)	−0.90 (−1.26, -0.53)*
Motility (% motile)	Reference	−4.63 (−11.16, 1.90)	−4.04 (−10.83, 2.76)	−10.87 (−17.74, -4.00)*
**CASA motion parameters**				
VSL (μm/s)	Reference	−1.30 (−4.08, 1.47 )	−1.93 (−4.87, 1.01)	−5.58 (−11.26, 0.10)
VCL (μm/s)	Reference	−1.62 (−5.15, 1.91)	−1.95 (−5.60, 1.70)	−8.48 (−11.97, -5.00)*
LIN (%)	Reference	−1.82 (−5.11, 1.46)	0.86 (−2.45, 4.16 )	−2.89 (−6.22, 0.44)
**DNA Fragmentation (%)**^b^	Reference	0.25 (−0.02, 0.52)	0.21 (−0.07, 0.49)	0.48 (0.20, 0.76)*

^a^Adjusted for age, smoking, alcohol drinking, BMI and abstinence time.

^b^ln-transformed.

*P <0.05 compared with the lowest sperm PAH-DNA adducts tertile (Tertile 1).

**Figure 2:**
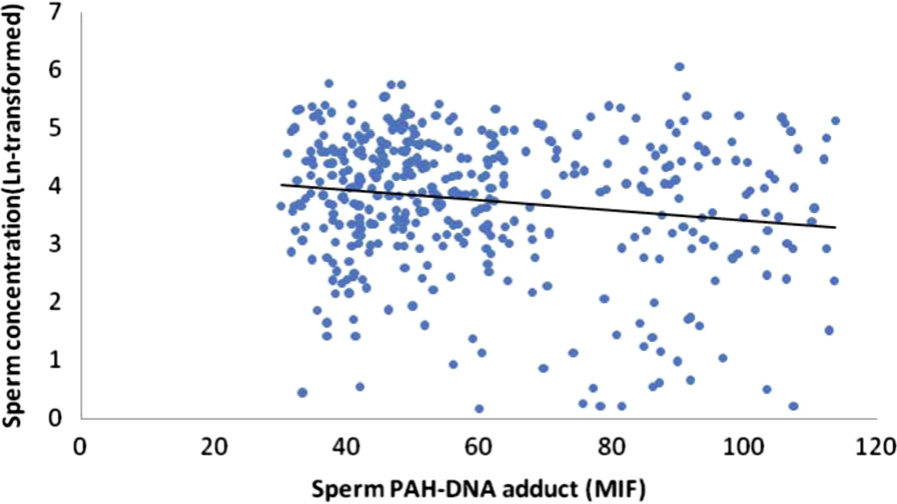
**Association between logarithm of the sperm concentration and sperm PAH-DNA adduct.** Adjusted for age, smoking, alcohol drinking, BMI and abstinence time, the PAH-DNA adduct levels were significantly associated with sperm concentration using a linear regression: beta coefficients = −0.632, *P* < 0.001.

**Figure 3:**
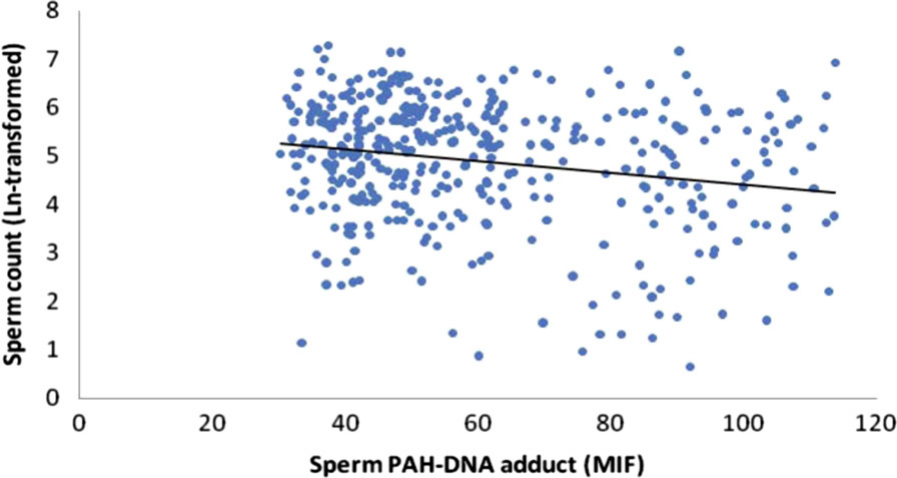
**Association between logarithm of the sperm count and sperm PAH-DNA adduct.** Adjusted for age, smoking, alcohol drinking, BMI and abstinence time, the PAH-DNA adduct levels were significantly associated with sperm count using a linear regression: beta coefficients = −0.632, *P* < 0.001.

**Figure 4:**
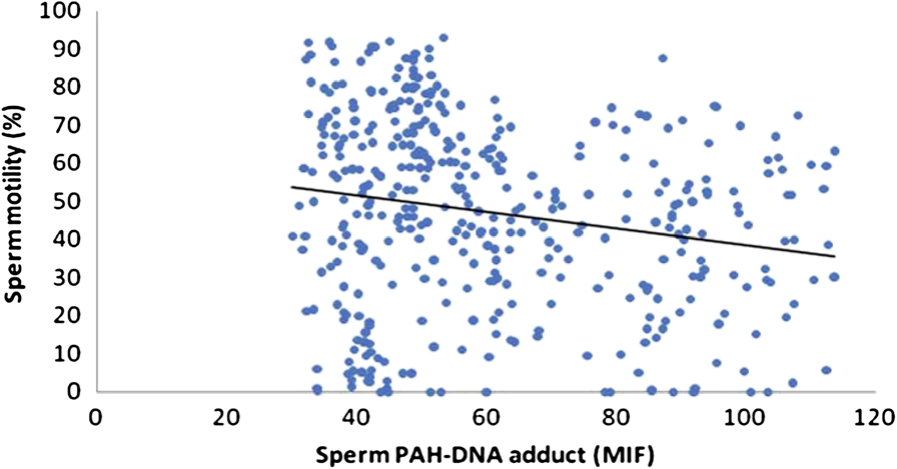
**Association between the sperm motility and sperm PAH-DNA adduct.** Adjusted for age, smoking, alcohol drinking, BMI and abstinence time, the PAH-DNA adduct levels were significantly associated with sperm motility using a linear regression: beta coefficients = −9.647, *P* = 0.012.

**Figure 5:**
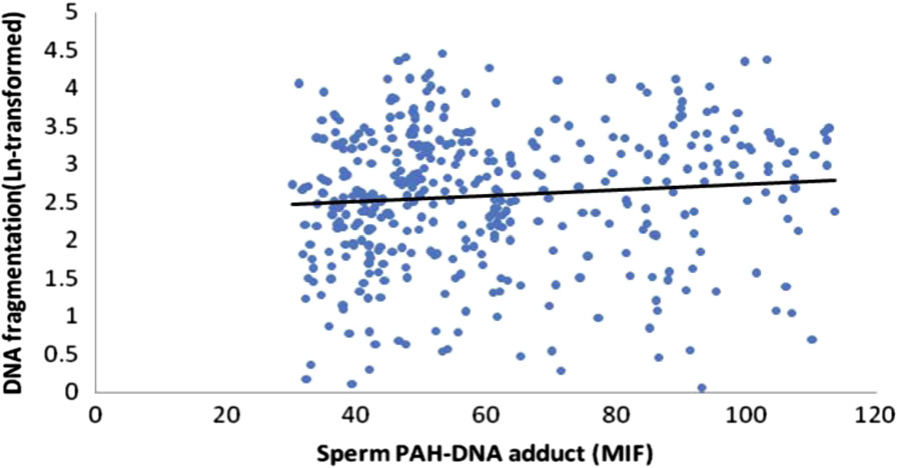
**Association between logarithm of the sperm DNA fragmentation and sperm PAH-DNA adduct.** Adjusted for age, smoking, alcohol drinking, BMI and abstinence time, the PAH-DNA adduct levels were significantly associated with sperm DNA fragmentation using a linear regression: beta coefficients = 0.130, *P* < 0.001.

The scatter plots were also used to represent the relationships between the PAH-DNA adducts levels and semen quality. As showed in Figures 2, 3, 4 and 5, significantly increased levels of PAH-DNA adducts were observed in subjects with high levels of sperm DNA fragmentation and lower sperm quality parameters.

## Discussion

Accumulating evidence suggest that sperm DNA integrity is essential for the accurate transmission of genetic information, and any form of sperm DNA damage may result in male infertility regardless of the number, motility and morphology of spermatozoa [25,26]. The clinical significance of sperm DNA damage lies in its association not only with natural conception rates, but also with assisted reproduction success rates [17,19].

Although the clinical significance of testing sperm DNA integrity has been clearly emphasized, the origin of DNA damage in spermatozoa is poorly understood. Accumulating evidence suggest that exposure to some environmental pollutions can induce DNA damage, either strand breaks or specific base modifications such as 8-oxoguanine. However, studies of sperm DNA damage have largely been limited to measurement of strand breaks or oxidative DNA damage [27], little is known about other types of DNA damage such as bulky DNA adducts in human sperm.

This study examined the associations between sperm PAH-DNA adducts and semen quality as well as sperm DNA fragmentation in adult men with non-occupational exposure to PAHs. Our suggestive findings of negative associations between sperm PAH-DNA adducts and poor semen quality were consistent with previous reports. In a Polish study, the ^32^P-postlabeling assay was used to assess the sperm PAH-DNA adducts in 179 males. The authors find significantly negative correlations between sperm concentration, sperm motility and sperm DNA adducts in patients with impaired fertility. In addition, the level of DNA adducts are 1.35-fold higher in the infertile group as compared to healthy individuals [28,29]. In another study carried out in Italy, PAH-DNA adducts are found to be associated with abnormalities of the head of sperm by immunofluorescence using fluorescent microscope [30]. Moreover, a study in assisted reproduction indicates a significantly negative correlation between sperm DNA adducts and fertilization rate during intracytoplasmic sperm injection (ICSI), which emphasizes the potential clinical significance of PAH-DNA adducts in human sperm [31].

In the present study, we used the immunofluorescent assay to detect sperm PAH-DNA adducts. Although the immunofluorescence methods were not as specific or sensitive as mass spectrometry and provide a semiquantitative measure of DNA adduct levels, the monoclonal antibody 5D11 we used has been extensively validated for tissue-based quantification of relative DNA adduct levels [32-35]. Immunohistochemistry assay used 5D11 antibody, which in cell culture studies has been shown to produce strongly correlated staining levels (r = 0.99) with the treatment dose of benzo(a)pyrene diol epoxide [36]. In view of the large sample size, the intensity of FITC fluorescence was detected by the flow cytometer rather than by fluorescence microscopy in our study. To assess the efficiency and concordance of flow cytometry and fluorescence microscopy, we applied these two approaches in a total of 46 human sperm samples. A good correlation was detected between the results as measured by flow cytometry and fluorescence microscopy [23].

We used stringent criteria to exclude subjects with adverse medical histories, drug treatment, occupational chemical exposures, and genetic risk factors, even Y chromosome microdeletion, which are related to semen quality and sperm DNA integrity. In multivariate linear regression models, most of the possible factors such as age, abstinence time, smoking and drinking status, BMI, and some lifestyles have been taken into account. These data and the relative large sample size can help us to better demonstrate the adverse effects of PAH-DNA adducts on semen quality. One limitation in our study needs to be addressed. We only selected infertile men who may be more “susceptible” to PAHs and the results should only be applied to that sort of population.

## Conclusions

Our finding showed that both increased sperm DNA fragmentation and decreased semen quality were associated with sperm PAH-DNA adducts among men from a clinical infertile population. The current research emphasizes the need of a better understanding of the relationship between environmental exposures and semen quality.

## Abbreviations

PAHs: Polycyclic aromatic hydrocarbons; TUNEL: Deoxynucleotidyl transferase -mediated dUTP nick end labeling; VCL: Curvilinear velocity; 1-OHP: 1-hydroxypyrene; VSL: Straight-line velocity; ICSI: Intracytoplasmic sperm injection; ROS: Reactive oxygen species.

## Competing interests

The authors have declared that no competing interests exist.

## Authors’ contributions

All authors have read the final version of the manuscript and are in agreement that the work is ready for submission to a journal. JGX took part in the planning of the study and the acquisition of data, conducted the statistical analyses and did most of the writing. GAH planned and designed the study, helped interpreting the results and took part in the preparation and reviewing of the manuscript. YLF and WSM, carried out the sperm DNA fragmentation analysis and helped to draft the manuscript. LJN, WL and ZSH carried out the sperm PAH-DNA adduct analysis. SLL helped planning the statistical analyses, interpreting the results and revised the statistical parts of the manuscript.

## Authors’ informations

Guixiang Ji and Lifeng Yan are joint first authors.
